# Measuring Adherence to Family‐Centred Goal Setting in Paediatric Rehabilitation: A Retrospective Chart Review

**DOI:** 10.1111/jpc.70454

**Published:** 2026-06-02

**Authors:** Armaghan Dabbagh, Shauna Kingsnorth, Joanne Maxwell, Lindsay Bosveld, Nada Khalaf, Katherine Maine, Emilie Nelson, Heather Colquhoun

**Affiliations:** ^1^ Department of Occupational Science and Occupational Therapy University of Toronto Toronto Canada; ^2^ Rehabilitation Sciences Institute University of Toronto Toronto Canada; ^3^ Bloorview Research Institute and Evidence to Care Holland Bloorview Kids Rehabilitation Hospital Toronto Canada

**Keywords:** family‐centred goal setting, family‐centred practise, gap measurement, goal setting

## Abstract

**Aim:**

Family‐centred goal setting (FCGS) is seen as fundamental to improving meaningful outcomes in paediatric rehabilitation. However, the degree to which FCGS is implemented in paediatric rehabilitation is unknown. This study aimed to develop and utilise a Chart Audit Tool to measure adherence to FCGS in a Canadian inpatient paediatric brain injury unit.

**Methods:**

In this retrospective chart review, *a FCGS Chart Audit Tool* was developed, piloted on 17 charts, and refined. The audit tool evaluated three categories (i.e., Partnership, Identification and Agreement) and eight subcategories. Charts were included of patients discharged between January and December 2023, with a minimum 14‐day stay and involvement from at least two rehabilitation professionals. After a 70% inter‐rater reliability was achieved, the audit tool was applied independently by one researcher to review 50 charts. Descriptive analyses were employed to calculate the adherence rates of rehabilitation professionals to the recommended practise of FCGS.

**Results:**

Overall, FCGS mean adherence expressed per chart was observed in 60% of the 50 charts reviewed across all categories (range of 33% to 80%). Individual mean adherence rates for the categories were 68% for Partnership, 70% for Identification and 66% for Agreement. Across the eight subcategories, mean adherence rates varied between 7% for the use of ‘family‐centred tools’ to 79% for ‘goals supporting family priorities.’

**Conclusions:**

Results of a chart review suggest that FCGS is being implemented most of the time but with areas for improvement. The novel creation of an audit tool to systematically quantify FCGS provides an important foundation for future research.

## Brief Points

- What is already known on this topic.
  ○ Family‐centred goal setting (FCGS) is a cornerstone of paediatric rehabilitation, promoting family priorities and meaningful outcomes, yet its consistent implementation remains uncertain.
- What this paper adds.
  ○ This study developed and utilised a novel Chart Audit Tool to systematically measure adherence to FCGS practises across three components: Partnership, Identification and Agreement.
  ○ Whilst documentation often reflected family involvement, the following opportunities for enhanced practise were identified: the use of structured family‐centred tools and documentation of shared recommendations.
  ○ The newly developed audit tool provides a systematic approach to evaluating FCGS implementation, paving the way for future adherence studies and targeted knowledge translation efforts to improve family‐centred care in paediatric rehabilitation.

## 1 Introduction

Family‐Centred Goal Setting (FCGS) is a collaborative process where a healthcare provider, a child, and their family work together to identify and agree on therapeutic goals, ensuring that these goals align with the family's priorities and values to achieve meaningful outcomes [1]. Engaging a multi‐disciplinary team to optimise the participation and motivation of both the child and their family through FCGS is widely regarded as best practise [2]. Research highlights numerous benefits of family‐centred practise across various paediatric settings and populations, including improved family self‐efficacy, enhanced motivation, better outcomes for children and increased parental satisfaction with both the process and the results [3, 4, 5]. Whilst FCGS is considered valuable in multiple areas of paediatric rehabilitation (i.e., occupational therapy, physical therapy, speech‐language pathology), including inpatient settings [6] the extent of its consistent implementation remains uncertain, with some studies indicating variability and inconsistency in its application [7].

Family‐Centred Goal Setting is supported by several theories, including Goal‐Setting Theory and Self‐Efficacy Theory [8]. These theories emphasise that effective goals should be specific, appropriately challenging and meaningful, whilst also requiring individuals to have confidence in their ability to achieve them and understand the consequences of success or failure. Despite the theoretical frameworks and characteristics identified for FCGS, significant inconsistencies remain in how it is applied in practise [9]. Often, the process is informal, with healthcare practitioners relying on ad hoc methods rather than adhering to standardised approaches [7]. These inconsistencies can diminish the effectiveness of FCGS, leaving its well‐documented benefits unrealized. Moreover, variations in training, resource availability and practitioner expertise further exacerbate these gaps, highlighting the need for tools and strategies that can support its application. To date, no universally accepted framework or gold‐standard tool exists to measure or guide FCGS comprehensively [1]. The absence of such a standardised tool limits the ability of healthcare practitioners to consistently evaluate and refine the goal‐setting process, creating a pressing need for further research and development in this area [2].

There has been a longstanding recognition of delays in translating research findings into practical application [10]. This issue, combined with the current focus on evidence‐based, cost‐effective and accountable health care, has heightened interest in addressing the discrepancy between what is recommended and what is being implemented in practise, also referred to as evidence‐to‐practise (E‐P) gaps. Measuring and addressing these E‐P gaps can play a significant role in ensuring optimal health care delivery and thus maximise health outcomes. A common approach to measure E‐P gaps is the chart audit [11]. Chart audits, reviewing and assessing health records using pre‐set criteria, offer a tangible record of the care provided and align with professional documentation standards [11].

Despite the importance of FCGS to paediatric practise, no studies have proposed and utilised a tool to measure the degree to which FCGS is currently being employed. Therefore, we designed this study with the following two objectives:

- *Objective 1:* To develop a Chart Audit Tool for the measurement of FCGS.
- *Objective 2:* To use the tool to measure the degree of adherence to FCGS practises within an inpatient paediatric acquired brain injury unit, specifically across physical therapy, occupational therapy and speech‐language pathology disciplines.

## 2 Methods

This was an observational retrospective chart review study. The degree to which FCGS was adhered to by rehabilitation professionals was determined by creating and utilising a Chart Audit Tool for FCGS. The study was approved through the institutional Research Ethics Board at the University of Toronto. Due to the number of charts accessed and the nature of the data collected, a waiver of individual patient/family consent was granted.

### 2.1 Study Sample and Participants

The study sample included 50 patient cases in a Canadian inpatient paediatric acquired brain injury unit. Charts were included that indicated (1) discharge from the facility between 1 January to 31 December 2023, (2) a minimum 14‐day length of stay; and (3) treatment by at least two of the three designated rehabilitation professionals. The timeframe (1 year) was chosen to be long enough to correct for monthly or unusual trends but not so long that the data would feel outdated or representative of a time when different hospital procedures might have been in place. The length of stay requirement was to ensure that the child was admitted long enough such that FCGS would have reasonably occurred and the requirement for at least two team members was due to our focus on rehabilitation team‐based FCGS. Charts were excluded if they indicated a pattern of frequent discharge/admission (e.g., if a child was often transferred short term to acute care) as it was not clear that through care or time FCGS was likely to occur.

### 2.2 Developing and Piloting the FCGS Chart Audit Tool

The FCGS Chart Audit Tool was developed through a collaborative effort between two researchers, one knowledge broker, one clinical occupational therapy manager and four occupational therapy students, with input from a practising occupational therapist and physical therapist.

The development process began by identifying three key categories—Partnership, Identification and Agreement—derived from the operational definition of FCGS as the partnership between a healthcare provider, a child, and their family in the process of identifying and agreeing upon therapeutic goals whilst respecting the client and family priorities and values to ensure meaningful outcomes [1]. The three categories were further refined into eight specific subcategories as follows:

- *Partnership (two subcategories):* Family and/or child present at sessions where goals were determined; Family and/or child is an active participant in their team.
- *Identification (four subcategories):* Healthcare professional notes contain documentation of family/child priorities for rehab; Documentation that healthcare professional recommendations are shared with family; Use of Family centred tools (e.g., COPM); Clear goal statement.
- *Agreement (two subcategories):* Goals support client/family priorities; Mutually decided upon or agreed upon plan from client/family and healthcare professional.

To assess each subcategory, a rating scale was developed with criteria informed by recommended FCGS practises [9]. The scale ranged from 0 to 3, where 0 indicated the absence of documentation of the desired practise, 1 represented below standard or inadequate charting of the desired practise, 2 reflected documentation that met expectations and 3 denoted exemplary charting that exceeded expectations. The tool was pilot tested on a sample of 17 charts to determine clarity and ease of use.

### 2.3 The Data Source and Extraction Process

Study charts were accessed using the internal database of the paediatric rehabilitation facility, for which access was granted. We looked at notes chronologically within each chart that were related to occupational therapy, physical therapy and speech‐language pathology. To minimise bias, a pilot exercise occurred with the first eight charts that included measures of reliability [12] and multiple research team discussions to identify and address sources of discrepancy. Once an inter‐rater reliability of > 70% was achieved, the remaining 42 charts were assessed by only one extractor.

Each chart was analysed using the *FCGS Chart Audit Tool* to assess for the presence of the three categories and eight subcategories. Data examination was limited to the first 30 days of a patient's stay. This was done to reflect initial goal setting as subsequent goal setting throughout the length of stay would look different for each client/family and it would have been difficult to know optimal progression for each patient. For charts that indicated COVID isolation in the first 30 days, an additional 10 days of chart review were added to accommodate. This ensured 30 days of active rehabilitation treatment were examined.

### 2.4 Statistical Analysis

Descriptive statistics were calculated to summarise the chart characteristics as well as the implementation of FCGS. A calculation for FCGS was done for each chart separately as well as for each of the categories and subcategories overall. For each aspect, the mean score and frequency percentage of charts reflecting the presence of FCGS were reported.

## 3 Results

### 3.1 Chart Characteristics

A total of 51 charts from the inpatient paediatric acquired brain injury unit were evaluated for inclusion in the analysis. Except for one chart, all charts met the inclusion criteria, representing cases discharged between 1 January and 31 December 2023, with a minimum 14‐day length of stay and involvement from at least two rehabilitation professionals. Of these, 16% (*n* = 8) underwent double extraction to calculate inter‐rater reliability, which yielded a percent agreement of 70%. Our sample comprised an equal distribution of female (50%, *n* = 25) and male (50%, *n* = 25) participants. The age range spanned 11 years, with a median age of 13 years. Participants were grouped into the following age categories: 0–4 years (*n* = 9), 5–9 years (*n* = 7), 10–14 years (*n* = 19) and 15–19 years (*n* = 15). The conditions in our sample included acquired brain injuries, neuromusculoskeletal conditions and/or symptoms (e.g., cerebral palsy, orthopaedic disorders, spinal cord injury) and developmental, psychological or learning disorders.

### 3.2 Objective 1‐ Developing the FCGS Chart Audit Tool

The FCGS Chart Audit Tool was refined through a pilot study involving 17 charts that were not included in the final set of 50 charts used for objective 2. During this phase, the research team adjusted the rating criteria for each item to improve clarity and applicability. The finalised tool utilised a 4‐point scale (0 to 3) to evaluate adherence for each item. A score of 3 indicated full adherence to FCGS practises, whilst a score of 0 reflected the absence of adherence. The total potential score across all items ranged from 0 to 24, providing a comprehensive measure of adherence to FCGS. The tool and its full scoring guide are available in a Supporting File.

### 3.3 Objective 2‐ Measuring Adherence to FCGS

When examining the total score of FCGS within each chart, audit scores ranged from 8 (33.3%) to 19 (79.8%) out of 24. Table 1 reports the adherence to the recommended practise of FCGS for each category and subcategory. The category of *Partnership* demonstrated a moderate level of adherence, with a mean adherence percentage of 68.0%. The highest‐performing subcategory was ‘Family as an active team participant’, with a mean score of 2.4 (78.7%), reflecting strong family involvement in the therapeutic team. However, ‘Presence at goal determination’ scored lower, with a mean of 1.7 (57.3%), indicating inconsistencies in ensuring families are present during critical decision‐making moments. The category of *Identification* had an overall mean adherence percentage of 69.8%, showing relatively strong implementation in documenting therapeutic goals. The subcategory ‘Clear goal statement’ performed well, with a mean score of 2.3 (78.0%), indicating that most goals were well‐defined and specific. Similarly, ‘Documentation of family/child priorities’ scored a mean of 2.2 (73.3%), highlighting efforts to align therapeutic goals with the preferences and values of the family. However, ‘Documentation that healthcare professional recommendations are shared with family’ and ‘Use of family‐centred tools’ were identified as weak points, scoring 1.5 (50.7%) and 0.2 (7.3%), respectively. The category of *Agreement* demonstrated a mean adherence percentage of 66.3%. The subcategory ‘Goals support family priorities’ achieved the highest mean score in this component, with 2.4 (79.3%), reflecting a strong alignment between documented goals and family‐identified needs. However, ‘Mutual agreement of plan’ was less consistently seen, with a mean score of 1.6 (53.3%).

**TABLE 1:** Adherence to the recommended practise of family centred goal setting.

Category	Mean score, out of 3	Mean percentage (%)
Partnership	—	68.0
Presence at goal determination	1.7	57.3
Family as an active team participant	2.4	78.7
Identification	—	69.8
Documentation of family/child priorities	2.2	73.3
Documentation of healthcare professional recommendations being shared	1.5	50.7
Use of family centred tools	0.2	7.3
Clear goal statement	2.3	78.0
Agreement	—	66.3
Goals support family priorities	2.4	79.3
Mutual agreement of plan	1.6	53.3

## 4 Discussion

This study was conducted as a retrospective chart review to determine the degree to which FCGS by rehabilitation professionals was occurring in an inpatient paediatric acquired brain injury unit. Adherence to FCGS components showed variability across charts, with total scores ranging from 33.3% to 79.8%. The Partnership component demonstrated moderate adherence (68.0%), with strong family participation but lower consistency in ensuring family presence during goal setting. Identification (69.8%) was well‐implemented overall, with clear goal statements and alignment with family priorities, but limited use of family‐centred tools highlighted a gap. Agreement (66.3%) reflected strong goal alignment with family priorities but less consistent documentation of mutual agreement, indicating areas for improvement in collaborative goal‐setting processes.

There were some subcategories where differences in adherence were surprising. Documenting healthcare professional recommendations as being shared with the family scored lower (50.7%) than documentation of the sharing of family‐child priorities (73%). It could be seen as unusual that healthcare professional recommendations were not shared as frequently as family/child priorities; however, the chart audit does not account for any nuances in how a healthcare professional, team, organisation or system approaches goal setting and its documentation. Documentation can occur by exception whereby concepts such as agreement are implied and documentation would occur only for disagreement or even when disagreement turned into agreement [13]. Documentation can be limited by a focus on procedures or task completion more than nuance and complexity [14] or reasoning and ‘thinking’ [15]. Whilst not the lowest adherence, presence at goal determination was also on the lower end (57.3%). Whilst we do not know for sure, this could be the kind of occurrence that is implied and not specifically documented. Goal setting itself is also complex [1] and may not lend itself well to discrete chart entry‐based analysis like in our Audit Tool.

Despite there being a presence of some degree of FCGS within all charts examined, and moderate levels of FCGS across categories, comprehensive FCGS was not found. Room for improvement was present in all areas. Our findings suggest that whilst families may be engaged in broader collaborative processes, there may be barriers preventing their full involvement at key stages of goal setting. Likewise, there may be barriers that impact the rehabilitation healthcare providers' ability to achieve FCGS. The limited use of family‐centred tools, such as structured assessments or collaborative planning aids, represents a significant gap, suggesting a need for enhanced resources and training to support clinicians in integrating these tools into practise. Whilst the use of these types of tools and outcome measures tends to be limited in rehabilitation in general [16, 17], a use rate higher than 7% seems reasonable to achieve. Some of the discrepancies highlight potential challenges in achieving consensus amongst families, children and healthcare providers. Contributing factors may include communication gaps, differing expectations or a lack of formal processes to document agreement. FCGS is crucial for delivering cohesive, client‐centred care [18]. Research indicates that patient outcomes improve when there is a unified approach to goal setting within the therapeutic team [19]. These findings highlight families who may not be receiving optimal FCGS and underscore the need for healthcare providers to refine and enhance clinical practises for this paediatric population. Other factors not addressed in the objectives of this study are worthy of consideration, including the ages of the children, family socioeconomic status and parental education levels. Understanding how issues such as these impact FCGS would be important for healthcare professionals who aim to optimise practise in this area.

To address the inconsistencies in the implementation of FCGS, there is a clear need for targeted knowledge translation (KT) efforts. KT strategies can bridge the E‐P gap by promoting the adoption of evidence‐based approaches, such as FCGS, into routine care. One effective KT method is audit and feedback [20], which involves systematically reviewing current practises and providing clinicians with feedback on their performance. This study serves as the audit component, highlighting areas where FCGS implementation may be lacking or inconsistent. When paired with timely and actionable feedback, this approach has been shown to improve healthcare practises by encouraging reflection, increasing awareness of performance gaps and motivating clinicians to adopt best practises [21]. Studies that examine the value of using audit data to improve practise would be a useful future approach.

### 4.1 Limitations

The limitations of this study include utilising a retrospective chart review to collect data, which may not capture the actual behaviour of FCGS. Chart audits can be a blunt instrument and likely do not provide the complete picture of the care delivered. A lack of documented engagement might be appropriate to any given chart due to factors such as emotional climate or availability of interpreters. However, all regulated healthcare providers are typically required by their regulatory bodies to document care; therefore, data within charts is a relatively good proxy for actual care. To quantify the degree to which FCGS was implemented, a chart audit tool had to be developed. Whilst we believe that the tool could be applied by any team wanting to measure FCGS adherence, it is important to consider if adaptations to context are required and piloting the tool is necessary. With more use of the tool, it is feasible it could become more developed and applicable across settings. We used a current definition of FCGS to develop the tool, but it is possible that additional elements of FCGS exist that our tool does not capture. Additionally, we chose a 4‐point scale for our tool as we were only able to describe four different levels of FCGS per subcategory, but it is possible that a 5‐ or 7‐point scale could be developed that would offer increased discrimination. We did not aim to examine the impact of child age on adherence. This would be a valuable future research direction, particularly given our sample ranged from 4 to 19 years of age. In addition, qualitative approaches with both children and parents would be advantageous to capture perspectives on the experiences of FCGS, and gain a better understanding of how, and how accurately, FCGS is captured in documentation.

## 5 Conclusion

Overall, FCGS appeared to be present in all charts, and by extension, in practise. However, FCGS varied significantly across subcategories, showing room for improvement. Whilst documentation often reflected family involvement, the use of structured family‐centred tools and documentation of shared recommendations represented opportunities for enhanced practise. The novel Chart Audit Tool to measure adherence to FCGS practises provides an approach to evaluating FCGS implementation, paving the way for future adherence studies and targeted knowledge translation efforts to improve family‐centred care in paediatric rehabilitation.

## Funding

The authors have nothing to report.

## Ethics Statement

This research project has been approved by the Research Ethics Board at the University of Toronto.

## Consent

The authors have nothing to report.

## Conflicts of Interest

The authors declare no conflicts of interest.

## Supporting information


**Data S1:** jpc70454‐sup‐0001‐SupinfoS1.docx.

**Data S2:** STROBE statement—checklist of items that should be included in reports of cross‐sectional studies.

## Data Availability

The data that support the findings of this study are available on request from the corresponding author. The data are not publicly available due to privacy or ethical restrictions.
