# Supplementary material for: Measuring Adherence to Family‐Centred Goal Setting in Paediatric Rehabilitation: A Retrospective Chart Review
Source: J Paediatr Child Health. 2026 Jun 2;62(8):1426–31. doi: 10.1111/jpc.70454 (PMC13480508; doi:10.1111/jpc.70454)
Supplement: Supplementary file 1 [file JPC-62-1426-s001.docx]

**Supplemental File**

Family-Centered Goal Setting (FCGS) Chart Audit Tool

**Participant ID: _________________________________**

**Scored By: _____________________________________**

**Date: __________________________________________**

| **Key Words** | **Categories for FCGS** | **Scoring** | | | | | | |
| --- | --- | --- | --- | --- | --- | --- | --- | --- |
|  |  | **0** | **1** | **2** | **3** | **Score Assigned** | | |
|  |  |  |  |  |  | **Rater 1** | **Rater 2** | **Conclusion** |
| **Partnership** | 1. **Family and/or child present at sessions where goals were determined.** | No documentation that family/child attended sessions where goals were determined. | Some sessions (<50%) where goals were determined included the family and/or child. | Most sessions (>50%) where goals were determined included both the family and/or child. | All sessions where goals were determined included both the family and/or child. |  |  |  |
|  | 2.  **Family and/or child is an active participant in their team.** | No documentation of family participation in any goal setting sessions. | Family concerns are documented, but no goals or plan discussed as a result – e.g., family stated “child is tired” or “unable to ascend stairs”. | Documentation on family sharing concerns and goals for rehab (e.g., family expressed desire to ascend stairs independently) some (but not all) goals/plans reflect this in at least 1 HCP. | Documentation on family sharing concerns and goals for rehab (e.g., family expressed desire to ascend stairs independently) goals/plans reflect this in all HCP. |  |  |  |
| **Identification** | 3.  **HCP notes contain documentation of family/child priorities for rehab** | No documentation of child/ family priorities. | In at least one HCP note, there is documentation of at least one child/ family concern, but their priorities for rehab are unclear. | In a team note or in at least two HCP notes, priorities for rehab of the child/family are documented - "X is important to me”, “I would like to work on X”. | In a team note or in all participating disciplines of HCP notes, priorities for rehab of the child/family are documented - "X is important to me”, “I would like to work on X”. |  |  |  |
|  | 4.  **Documentation that HCP recommendations are shared with family** | No areas for improvement and/or recommendations are identified and shared with family. | Some areas of rehabilitation improvement identified in chart, but no documentation suggesting that recommendations for those areas of improvement were shared with the family. | Clear areas of rehabilitation improvement are identified in chart and there are some (but not all) documentations suggesting that some (but not all) recommendations for those areas of improvement were shared with the family. | Clear areas of rehabilitation improvement are identified in chart and there is documentation suggesting that all recommendations for those areas of improvement were shared with the family. |  |  |  |
|  | 5.  **Use of Family centered tools (e.g., COPM)** | No use of family-centered goal setting tools. | There was some suggestion of a family centered tool being used, but based on documentation it was unclear for certainty whether it was, or which one. | Family centered tool was used, but family involvement is unclear through its use or not complete. | Family centered tools are complete and suggests family involvement. |  |  |  |
|  | 6. **Clear goal statement** | No goal stated. | General goal areas stated but no clear goal. | Some priority goals are clearly stated but not all. | Prioritized goals are clearly stated. |  |  |  |
| **Agreement** | 7.  **Goals support client/family priorities** | Goals stated do not support client/family priorities | HCPs incorporate some client / family observations into goals. | HCPs incorporate most, but left out some key client / family priorities into goals but do not reflect all of them. | Prioritized goals support client/family priorities. |  |  |  |
|  | 8.  **Mutually decided upon or agreed upon plan from client/family and HCP** | No documentation of family agreeing upon goal/plan or documentation of disagreement. | Documentation of HCP sharing concerns and plan with family, while goals reflect previous discussions, no documentation of family agreeing on goals. | Documentation of HCP sharing concerns and plan with family. Statement that child/family agreed to some but not all goals, or clear implicit agreement based on rehab goals reflecting patience concerns / goals. | Documentation of HCP sharing concerns and plan with family. Statement regarding agreement to all goals from child and family "e.g., child and family agreed upon goals". |  |  |  |
| Total Score | | | | | |  |  |  |
